# Origin, impacts, and potential solutions to the fragmentation of the Mexican health system: a consultation with key actors

**DOI:** 10.1186/s12961-023-01025-2

**Published:** 2023-07-31

**Authors:** Octavio Gómez-Dantés, Laura Flamand, Diego Cerecero-García, Mariana Morales-Vazquez, Edson Serván-Mori

**Affiliations:** 1 Center for Health Systems Research, The National Institute of Public Health, 62100 Cuernavaca, Morelos Mexico; 2 Center for International Studies, College of Mexico, A.C., Mexico City, Mexico; 3 University of Toronto, Toronto, Canada

**Keywords:** Health system fragmentation, UHC, Mexican health system

## Abstract

**Background:**

One of the central debates in health policy is related to the fragmentation of health systems. Fragmentation is perceived as a major obstacle to UHC. This article presents the results of a consultation with a group of actors of the Mexican policy arena on the origins and impacts of the fragmentation of the Mexican health system.

**Methods:**

We used a consultation to nine key actors to collect thoughts on the fragmentation of the Mexican health system. The group included national and local decision makers with experience in health care issues and researchers with background in health systems and/or public policies. The sessions were recorded, transcribed verbatim and analyzed thematically.

**Results:**

Participants defined the term ‘fragmentation’ as the separation of the various groups of the population based on characteristics which define their access to health care services. This is a core characteristic of health systems in Latin America (LA). In general, those affiliated to social security institutions have a higher per capita expenditure than those without social security, which translates into differential health benefits. According to the actors in this consultation, fragmentation is the main structural problem of the Mexican health system. Actors agreed that the best way to end fragmentation is through the creation of a universal health system. Defragmentation plans should include a research component to document the impacts of fragmentation, and design and test the instruments needed for the integration process.

**Conclusions:**

First, health system fragmentation in Mexico has created problems of equity since different population groups have unequal access to public resources and different health benefits. Second, Mexico needs to move beyond the fragmentation of its health system and guarantee, through its financial integration, access to the same package of health services to all its citizens. Third, defragmentation plans should include a research component to document the impacts of fragmentation, and design and test the instruments needed for the integration process. Fourth, defragmentation of health systems is not an easy task because there are vested interests that oppose its implementation. Political strategies to meet the resistance of these groups are an essential component of any defragmentation plan.

## Background

Most health systems in Latin America (LA) are fragmented. According to Bossert et al., fragmentation implies the coexistence of several health care delivery subsystems to provide services to different population groups, financed by various funding pools, and run by specific rules for accessing financial resources and health benefits [1].

There is evidence that suggests that fragmentation increases inequalities as the members of groups with economic disadvantages are often assigned less public resources, receive fewer services of poorer quality, and, in general, present worst health outcomes [2]. Fragmentation also increases the propensity of catastrophic expenditure (i.e., the health expenditure in excess of 30 per cent of disposable income, which, in turn, is defined as total family income minus expenditure in food). One comparative analysis of 12 LA countries found that the proportion of households with catastrophic health expenditures in these nations ranged from 1 to 25% [3]. Catastrophic health expenditures were more prevalent among those living in rural areas, the poor, those living in households with older adults, and those without social security.

Several countries in the LA region are involved in some type of reform to expand access to health care and improve financial protection through, among other things, the reduction of fragmentation [4, 5]. Although these reforms vary on the proposed sources of funds and the role of public and private health care providers, most of them aim at increasing pooled funding and universal health coverage (UHC). It is crucial to follow these reforms and assess their impact.

In this article we present the results of a consultation with a group of key actors of the Mexican policy arena on the origins and impacts of the fragmentation of the Mexican health system. It also provides some insights into its possible solutions and suggests future research areas to further document this problem and contribute to the design of policies to reduce fragmentation to provide the same health benefits, with the same level of quality and under the same rules to all the Mexican population.

## The Mexican health system

**Fig. 1:**
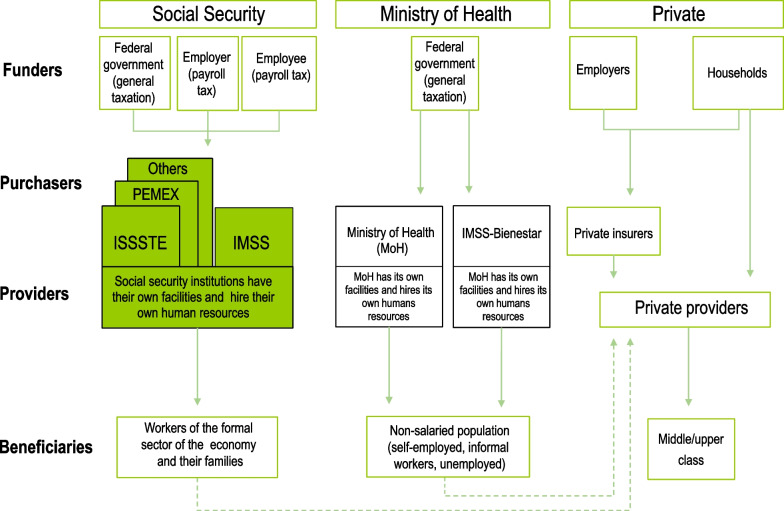
The Mexican health system in 2022

The Mexican health system was created in 1943. It has a public and a private component (Fig. 1) [6–8﻿]. The public component includes two basic subsystems: (i) social security [Mexican Institute for Social Security (IMSS), Social Security and Services Institute for Civil Servants (ISSSTE), Social Security Institute for Oil Workers (Pemex), Social Security Institute for the Armed Forces (Sedena), Social Security Institute for the Navy (Semar)], which provides comprehensive health care to the population with contributive social security (56.6 million people), and (ii) the federal and state ministries of health and IMSS-*Bienestar* (IMSS-B)—small institution managed by IMSS which until recently provided basic care to the rural poor—, which provide health care to those lacking contributive social security (69.4 million people). In addition, the private sector component offers health care to the population with the capacity-to-pay in facilities offering services on a for-profit basis.

The social security subsystem in Mexico has essentially remained unchanged since the establishment of IMSS in 1943 and the other social security agencies in the 1960s. The component responsible for providing health care to those without social security, in contrast, has changed considerably in the past 20 years.

In 2003, the Mexican Congress approved a reform to provide social protection in health to those lacking social security through the System for Social Protection in Health (SSPH) and its financial vehicle, *Seguro Popular* (SP) [9]. SP guaranteed access to 294 essential services and 66 high-cost interventions. In 2015, over 50 million people were affiliated to SP.

After the election of December 2018, the new federal administration decided to dismantle SP and establish in its place the Health Institute for Welfare (*Instituto de Salud para el Bienestar* or INSABI). The new institute, launched in January of 2019, would negotiate agreements with Mexico’s 32 states to provide health services to the population lacking contributive social security, recentralizing the delivery of care within the MoH [10]. However, to date, only 26 states have joined INSABI, and six remain decentralized [11]. In terms of benefits, INSABI would be legally responsible for providing outpatient and general hospitalization services, but not specialty services [12].

The initial results of INSABI’s implementation were disturbing. By 2020, the number of people in Mexico without access to health services had grown to 35.7 million, an increase of 15.6 million in two years. According to the National Council for the Evaluation of Social Development Policy, most of them did not know they were entitled to free services and medicines in INSABI [13].

In February of 2022, in response to the implementation problems of INSABI, the federal government announced its intention of upgrading IMSS-B by strengthening its infrastructure and human resources in order to provide health care to the population without social security [14]. This process, branded as ‘federalization of health care’, includes the incorporation of the local hospitals presently managed by the state ministries of health to IMSS-B. The new role of INSABI within this process of federalization remains unclear. INSABI was finally dismantled in May 2023.

## Materials and methods

The research question we intend to answer in this study is the following: what are the origins, impacts, and potential solutions to the fragmentation of the Mexican health system. To answer this question, we used a consultation with key actors to gather information and thoughts on the fragmentation of the Mexican health system. According to some authors, this type of consultation allows citizens and stakeholders to actively participate in policy design and implementation, and increases the capacity of governments to solve societal problems in a legitimate way [15]. It also gives “voice to multiple perspectives and different interests, allowing for more thoughtful decisions that take a broader view of those who will benefit or be harmed by an action [16].”

Nine actors were invited to participate in this consultation. The purpose of the sampling was to gather a group that included national and sub-national decision makers with experience in health care issues and researchers with background in health systems and/or public policy research both from public and private institutions. The selection of the sample was bases on convenience, assuming that the number of selected actors and their profile would meet the goals of the consultation.

The selected participants included two women and seven men. Six of them are physicians, all of them with public health training, and two are social scientists with training in health policy. The group also included an actuary. Three of the actors had been high-level decision makers at the federal Ministry of Health (MoH) of Mexico in previous administrations. Two of them are active decision makers, one at the MoH of Mexico City and the second one at the MoH of one state of the country. Finally, four of the actors are presently working as researchers on health policy issues. Three of them work at public academic institutions and one of them at a private university. At the end of the discussion section, we describe a major limitation of the study related to the selection of actors.

All selected actors were invited to participate in a semi-structured interview on the fragmentation of the Mexican health system. They all accepted and agreed to have their session recorded. They were told that their names would not appear in the final report of the consultation, but that a general description of their professional profile would be included. For confidentiality purposes, we indicated that we would destroy the recordings once the analysis was concluded.

The nine actors received in advance a set of ten questions related to the following topics: (i) definition, origin, and effects of the fragmentation of the Mexican health system; (ii) potential solutions to fragmentation and the political feasibility of these solutions; and (iii) type of research needed to document and reduce the fragmentation of this system. Participants were interviewed by phone—in a call that lasted, on average, 45 min—during the first two weeks of May of 2021. Each interview was transcribed by a research assistant, and the transcripts were reviewed by two of the authors. We coded the answers around the three main topics described above, which, according to the literature, cover all key issues related to health sector reforms aimed at defragmentation [17, 18].

## Results

### Health system fragmentation—definition

Most participants in this consultation defined the term ‘fragmentation’ in similar terms: as the separation of the various groups of the population based on certain characteristics which define their access to health care services. According to them, in Mexico and many countries of the Latin American region, the type of occupation is the key variable to access comprehensive health care and, thus, explains the fragmentation of health systems.

### Origins of health system fragmentation in Mexico

According to three of the actors of this consultation, during the twentieth century, in many countries of Latin America, for political and/or economic motives, certain populations were considered priority groups and, for this reason, had access to privileged health care. In Mexico, the workers of the formal private sector of the economy were considered a priority group and a special social security agency, IMSS, was created for them in 1943. This ‘privilege’ was then extended to those in the formal public sector of the economy. In the 1960s, social security agencies were created for civil servants (ISSSTE), oil workers (Pemex) and the armed forces (Sedena and Semar). For a short period of time, railroad workers and workers of the electric sector also counted with their own social security institutions. During this period (1943–2003), the population without social security (informal workers, self-employed, unemployed, and those outside the labor market) received health care as a public charity through services provided mostly by the MoH. According to the original design of the Mexican health system, eventually, all the Mexican population would be part of the formal sector of the economy and would be affiliated either to IMSS, ISSSTE, Pemex, Sedena or Semar. One of the researchers stated:

*“Unfortunately, this didn’t occur, due to the precariousness of the labor market, and today more than half of the total population works in the informal sector of the economy.”*

The result is a system with various components—and various subcomponents—with differences in financial resources per capita. In general, those affiliated to social security institutions have a higher per capita expenditure than those without social security, which translates into differential health benefits and health conditions.

An actor in this consultation stated that fragmentation:

“[…] is also an expression of poor stewardship, since one of its main subfunctions is the definition of access to health care services, which in a democracy should be universal.”

### Impacts of health system fragmentation in Mexico

According to one of the actors in this consultation, “[…] fragmentation is the main structural problem of the Mexican health system,” and all actors indicated that the most relevant negative effect of fragmentation is that the various components of the system are unequal. The allocation of financial resources per capita for each component is different, and so are the health benefits and the quality of care that the different population groups receive. This also creates disparities in the levels of financial protection across its pieces. One of the actors, a former high official of the MoH, stated the following:

*“Fragmentation implies the existence of privileges associated to the political strength of the groups that receive them. These privileges generate equity problems. Depending on the magnitude of the benefited segments, the aggregate effect of fragmentation on inequity can vary. In Mexico, given the size of the salaried population (around half of the total population), the effect of fragmentation on national inequity is huge.”*

The second negative effect is inefficiency, especially in those national systems that have various health care delivery structures, such as Mexico. In each subsystem, infrastructure tends to be under-utilized [19].

All actors agreed that the main loser of fragmentation is the uninsured population, which includes low-wage workers employed in unstable labor positions. Finally, one actor mentioned that there is also a problem of rights differentiation, which rips the social fabric:

*“Instead of acting as the great equalizer by providing the same package of health care services with the same quality to all the population, the health system provides services to the various population groups using different criteria. The salaried population has the right to comprehensive health care, while the non-salaried population receives essential health services as public charity.”*

All actors stated that health system fragmentation in Mexico has generated no positive effects, “[…] not even the benefits of a potential competition among different health care providers,” since all health services are provided through monopolies to which non-affiliated individuals have no legal access.

### Solutions to health system fragmentation

According to most actors, in Mexico, the first step toward defragmentation was the creation, in 2003, of the System of Social Protection in Health (SSPH) for the population without social security, which included SP, its financial vehicle. This was an analogous insurance scheme—with a similar financial architecture—to that of social security agencies. This characteristic would eventually favor the integration of the Mexican health system. In the opinion of those interviewed, SP did not close the gaps among the various health care subsystems in Mexico, but it reduced them considerably. SP was able to reduce the financial and health benefits gaps between those with contributive social security and those without [9].

During the period of implementation of SP, health services portability was also established, especially for high-cost interventions, which included a list of health services with their price-catalogues which facilitated transactions among health care agencies. The most sophisticated integration mechanism would have been a universal health-card, which was not created.

Most actors agreed that the best way to end fragmentation is through the creation of a universal health system. One of them mentioned that:

*“[…] it should be ‘universal’ in two senses: it must be a system that covers everybody —and a fragmented system can cover everybody—, but with the same set of health benefits and with services that have the same quality. This implies the creation of single financial fund and universal access to all health facilities, eliminating the legal barriers that exist to date.”*

There was consensus around the need for a system that decouples labor status from access to health care, with a universal public insurance well financed with general taxes, and a plurality of health care providers, a health system with clear, common rules, a solid set of incentives, and efficient articulation mechanisms. One of the actors added:

*“One possibility is the reorganization of the health system by functions (financing, delivery, stewardship), where all functions are applied equally to all population groups.”*

Two actors stated that there is no space for a public monopoly responsible for the delivery of services, since competition contributes to quality and efficiency, and guarantees the freedom to choose the provider of care.

### Feasibility of solutions to health system fragmentation

Various actors mentioned that the present federal administration, which has control over the Congress, missed a huge opportunity to create a non-fragmented, pluralistic system, like that of the UK or Canada. Instead, the new administration initially opted for the creation of another public monopoly, INSABI.

Three actors mentioned that INSABI left the labor-based structures of the Mexican health system, which support its fragmentation, untouched, and has demolished all mechanisms intended to de-fragment it.

*“The Mexican health system is way behind what it was in 2018. At the end of the past administration, there were conditions that could help reduce the fragmentation of the system, but INSABI has strengthened it.”*

Two actors stated that IMSS, ISSSTE and Pemex bureaucracies and their unions have stood against the defragmentation of the health system. Since the creation of these agencies, through political support to an authoritarian regime headed by a single political party that ruled Mexico for over 70 years, the workers of these institutions have gained privileged labor conditions which are placing enormous financial pressure on their institutions. One of the actors added:

*“IMSS, ISSSTE and Pemex workers have consistently opposed all integration initiatives for fear of losing their privileges, while their unions have opposed them for fear of losing control over their affiliates.”*

Most actors agreed that there are no chances of moving towards integration nowadays. One of them stated that *“there is a need for a political change in order to stop the erosion of the national health system.”*

### Research on health system fragmentation

Various actors mentioned that health systems research should document the recent evolution of the allocation of public resources in Mexico to the different subsystems to know if the gaps are closing or widening. It should also measure the effects of these tendencies on health conditions and levels of financial protection.

Research should also take advantage of the introduction of new policies to compare the levels of access to health care and the health of the populations that are receiving the ‘benefits’ of INSABI (the population of the 26 states that joined this agency) with the levels of access to health care and the health of the populations that are not receiving them (the population of the six states that decided not to join INSABI).

In addition, research should measure the changes in indicators (health conditions, financial protection) associated to the disappearance of SP, which guaranteed access to 66 high-cost interventions, including treatment for cancer in children and treatment for cervical and breast cancer.

Efforts should also be devoted to costing the package of health benefits that could be provided to all Mexicans in a scenario of UHC and to the testing of measures to reduce inefficiency—duplications, managerial ineffectiveness—and liberate resources to expand and improve the delivery of health care services.

Operative health system research is also needed to test, through pilot interventions, the feasibility of delivery models that favor freedom of choice and competition among providers.

Finally, according to one of the actors, who has long-time experience in health systems research, comparative international research is required to know how other countries were able to de-fragment their systems. This implies the review not only of the organizational and financial measures taken by other countries to de-fragment their system, but also the political strategies used to meet the resistance of those groups opposing integration. According to this actor, “*[…] useful lessons will probably come not from the Latin American region but from Mediterranean/European countries, such as Greece, Italy, Portugal, and Spain […].*”

## Discussion

Health systems in Latin America have a shared history [20]. They were mostly created in the 1930s and 1940s. They included both the private and public sectors. The public sector typically included a well-resourced contributive social security component offering comprehensive health care on a rights basis, and a MoH component serving those without social security with essential health services of low quality provided on a public charity basis.

According to most actors interviewed for this article, the Mexican health system represents the typical fragmented model with the separation of population groups based on labor status. Despite the increase in access to comprehensive health care services of the non-insured population achieved in the past 20 years and its positive impact on health conditions and financial protection levels, fragmentation in Mexico is perceived as a barrier to the fulfillment of the right to the protection of health enshrined in Article 4 of the Mexican constitution [21]. The reason is that the two basic groups of the population (with and without social security) have unequal access to public resources for health per capita and different health benefits. This has generated gaps both in health conditions and levels of financial protection.

Various actors in this consultation stated that SP, active in the period 2004–2018, reduced the gaps in resources and health benefits between social security agencies and the institutions providing health care to the uninsured population, and created the financial conditions for the eventual integration of the Mexican system [22, 23]. In contrast, INSABI, which substituted SP in 2019, has reinforced the fragmented nature of the Mexican health system by extending the gaps in resources and benefits between social security agencies and those institutions providing health care to the population lacking social security [24]. The fragmented nature of the Mexican health system is being further boosted by the strengthening of IMSS-B, a small health care institution previously responsible for providing health care to the rural poor, mostly uninsured.

The main conclusion from those consulted—consistent with what various analysts in the region have discussed—is that Mexico needs to move beyond the fragmentation of its health system and guarantee, through its financial integration, regular access to the same package of comprehensive health services to all its citizens in order to reach UHC [13, 25–28]. This would eventually contribute to welfare and economic growth. This is also a recommendation for any country with a fragmented health system looking to provide access to comprehensive health services to all its population. The poor are considered the most vulnerable population among those with no social security. These efforts should be complemented by focused interventions to protect vulnerable populations, such as women, girls, indigenous people, the LGBTI+ community and those geographically isolated.

According to the results of this consultation, the adjective ‘universal’ in the term UHC means essentially two things: (i) access to comprehensive health care to all, regardless of their labor status and (ii) access to the same package of health benefits, which should be provided with the same level of quality. This implies the design of reforms intended to create a system with a single public financial fund —in order to guarantee freedom of choice and competition, which foster quality—and a plural delivery of health care services, with the MoH in charge of stewardship. Most actors agreed that health systems should try to move away from health care services monopolies which are, by definition, costly, and inefficient, and leave no room for the selection of the provider of care.

All actors also agreed that defragmentation plans should include a health system research component to document the impacts of fragmentation—in the distribution of public resources and services, health conditions, and financial protection—, design the organizational and financial instruments needed for the integration process, and test the delivery models that could be eventually used by a universal health system to guarantee a successful plural delivery of health care services.

A final conclusion of this consultation is that defragmentation of health systems is not an easy task because there are vested interests that oppose its implementation, most notably the workers of social security agencies and their unions. These groups oppose integration of fragmented health systems because they fear they could lose their labor and political privileges. Detailed political strategies to meet the resistance of these groups are an essential component of a defragmentation plan.

This paper has an important limitation related to the selection of actors. It did not include actors who, for various reasons, oppose the integration of the Mexican system, especially representatives from the main social security agencies. Actors from the private sector were also excluded, and they could provide interesting opinions about the need for integration of the health system and other possible solutions to move towards UHC, such as the possibility of developing public–private partnerships to expand access to comprehensive health care.

## Conclusions

In the opinion of the actors interviewed, the five main conclusions of this consultation are the following:

1. Health system fragmentation in Mexico has created serious problems of equity due to the fact that the two basic population groups (population with and without social security) have uneven access to public resources for health per capita and different health benefits.
2. Mexico needs to move beyond the fragmentation of its health system and guarantee, through its financial integration, access to the same package of comprehensive health services to all its citizens to reach UHC.
3. Defragmentation plans should include a health system research component to document the impacts of fragmentation, and design and test the instruments needed for the integration process.
4. Defragmentation of health systems is not an easy task because there are vested interests that oppose its implementation. Detailed political strategies to meet the resistance of opposing groups are an essential component of any defragmentation plan.

## Data Availability

The data analyzed during the current study are available from the corresponding author on reasonable request.
